# An efficient strategy to recellularization of a rat aorta scaffold: an optimized decellularization, detergent removal, and Apelin-13 immobilization

**DOI:** 10.1186/s40824-022-00295-1

**Published:** 2022-09-22

**Authors:** Saba Fooladi, Sanaz Faramarz, Shahriar Dabiri, Abdolmohammad Kajbafzadeh, Mohammad Hadi Nematollahi, Mehrnaz Mehrabani

**Affiliations:** 1 Student Research Committee, Kerman University of Medical Sciences, Kerman, Iran; 2 Department of Pathology, Pathology and Stem Cells Research Center, Afzalipour Medical School, Kerman University of Medical Sciences, Kerman, Iran; 3 Pediatric Urology and Regenerative Medicine Research Center, Gene, Cell and Tissue Research Institute, Children Hospital Medical Center, Tehran University of Medical Sciences, Tehran, Iran; 4 Physiology Research Center, Institute of Neuropharmacology, Kerman University of Medical Sciences, Kerman, Iran; 5 Department of Clinical Biochemistry, Kerman University of Medical Sciences, Kerman, Iran

**Keywords:** Apelin, Vascular tissue engineering, Decellularization, Recellularization, SDS, Triton X-100

## Abstract

**Background:**

Tissue engineering of native vessels is an alternative approach for patients with vascular disease who lack sufficient saphenous vein or other suitable conduits for autologous vascular graft. Moreover, the harvest of vessels prolongs the surgical procedure and it may lead to the morbidity of donor site in elder patients: therefore, it seems that the use of tissue-engineered vessels would be an attractive and less invasive substitute for autologous vascular grafts. Apelin-13 plays a pivotal role in cell proliferation, survival, and attachment; therefore, covalent attachment of apelin-13 to the acellular scaffolds might be a favorable approach for improving recellularization efficacy.

**Methods:**

In the present study, the decellularization process was performed using various detergents. Afterward, the efficacy of decellularization procedure was evaluated using multiple approaches including assessment of DNA, hydroxyproline, and GAG content as well as Masson’s trichrome and orcein staining used for collagen and elastin determination. Subsequently, the scaffold was bioconjugated with apelin-13 using the EDC-NHS linker and acellular scaffolds were recellularized using fibroblasts, endothelial cells, and smooth muscle cells. SEM images and characterization methods were also used to evaluate the effect of apelin-13 attachment to the acellular scaffold on tissue recellularization. We also developed a novel strategy to eliminate the remnant detergents from the scaffold and increase cell viability by incubating acellular scaffolds with Bio-Beads SM-2 resin. Testometric tensile testing machine was also used for the assessment of mechanical properties and uniaxial tensile strength of decellularized and recellularized vessels compared to that of native tissues.

**Results:**

Our results proposed 16-h perfusion of 0.25% sodium dodecyl sulfate (SDS) + 0.5% Triton X-100 combination to the vessel as an optimal decellularization protocol in terms of cell elimination as well as extracellular matrix preservation. Furthermore, the results demonstrated considerable elevation of cell adhesion and proliferation in scaffolds bioconjugated with apelin-13. The immunohistochemical (IHC) staining of CD31, α-SMA, and vimentin markers suggested placement of seeded cells in the suitable sites and considerable elevation of cell attachment within the scaffolds bioconjugated with apelin-13 compared to the non-bioconjugated, and decellularized groups. Moreover, the quantitative analysis of IHC staining of CD31, α-SMA, and vimentin markers suggested considerable elevation in the number of endothelial, smooth muscle, and fibroblast cells in the recellularized scaffolds bioconjugated with apelin-13 group (1.4% ± 0.02, 6.66% ± 0.23, and 9.87% ± 0.13%, respectively) compared to the non-bioconjugated scaffolds (0.03% ± 0.01, 0.28% ± 0.01, and 1.2% ± 0.09%, respectively) and decellularized groups (0.03% ± 0.007, 0.05% ± 0.01, and 0.13% ±0.005%, respectively). Although the maximum strain to the rupture was reduced in tissues decellularized using 0.5% SDS and CHAPS compared to that of native ones (116% ± 6.79, 139.1% ± 3.24, and 164% ± 8.54%, respectively), ultimate stress was decreased in all decellularized and recellularized groups. Besides, our results indicated that cell viability on the 1st, 3rd, and 7th day was 100.79% ± 0.7, 100.34% ± 0.08, and 111.24% ± 1.7% for the decellularized rat aorta conjugated with apelin-13, which was incubated for 48-h with Bio-Beads SM-2, and 73.37% ± 7.99, 47.6% ± 11.69, and 27.3% ± 7.89% for decellularized rat aorta scaffolds conjugated with apelin-13 and washed 48-h by PBS, respectively. These findings reveal that the incubation of the scaffold with Bio-Beads SM-2 is a novel and promising approach for increasing cell viability and growth within the scaffold.

**Conclusions:**

In conclusion, our results provide a platform in which xenograft vessels are decellularized properly in a short time, and the recellularization process is significantly improved after the bioconjugation of the acellular scaffold with apelin-13 in terms of cell adhesion and viability within the scaffold.

**Graphical Abstract:**

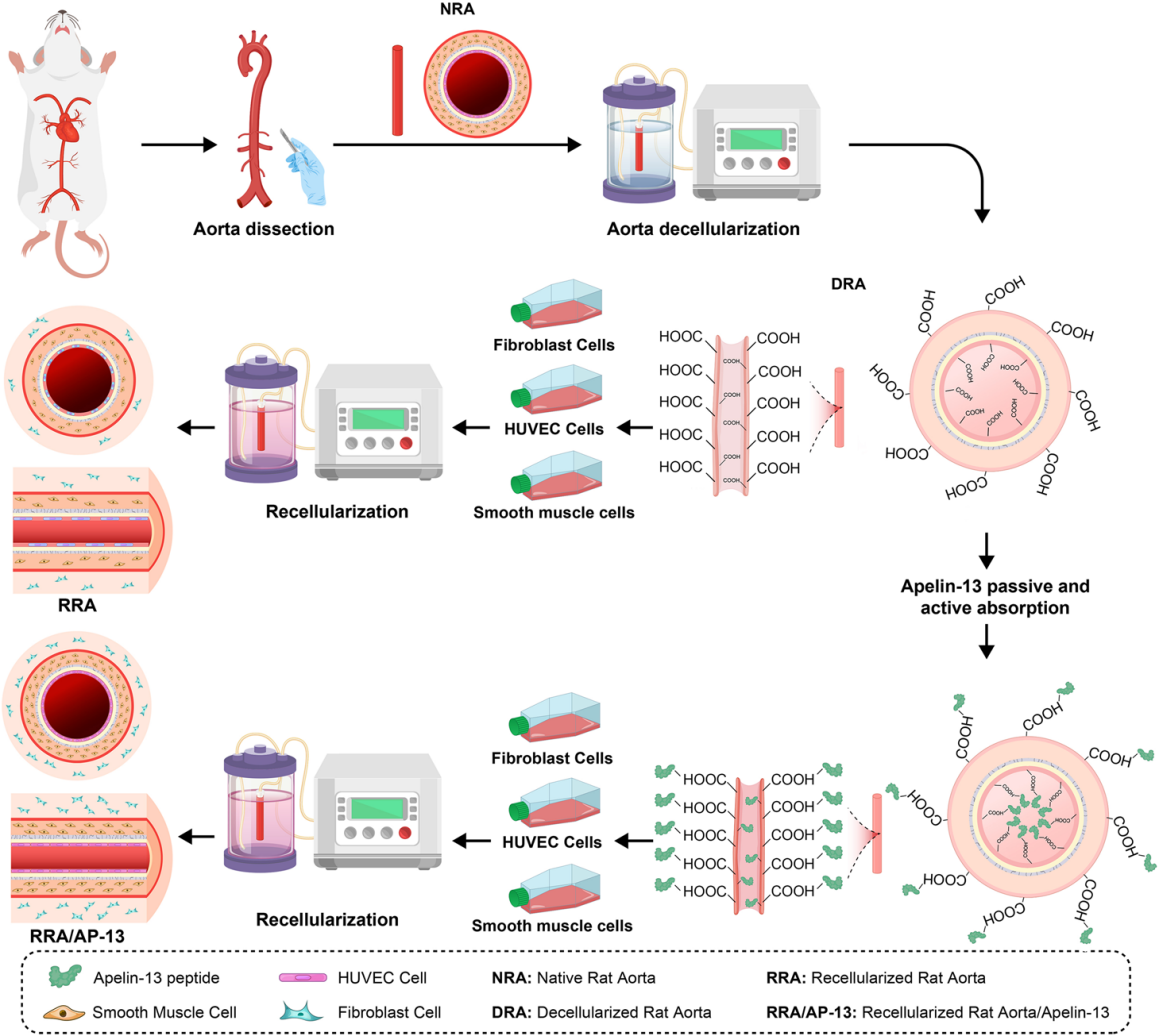

## Introduction

Cardiovascular disease is known as a common cause of death all around the world, and autologous vascular graft is considered the gold standard treatment for patients with advanced vascular diseases [1–3]. However, the availability of healthy and suitable vessels for autologous grafts is limited, and dissecting these vessels from the patient may lead to several complications; therefore, it seems that in vitro vascular engineering is a worthwhile option for addressing this clinical demand [4]. While the development of suitable tissue-engineered vessels is of great importance for clinical transplantation, there is a gap regarding the provision of appropriate vascular scaffolds and the most effective recellularization approaches [5]. Recently, the tissue engineering field of study has suggested using alternative scaffolds, including synthetic vascular grafts, to rectify this problem. However, none of these scaffolds fulfill the requirements of clinical application, including biocompatibility, as well as suitable mechanical and structural properties similar to native vessels. Therefore, decellularization of allogenic or xenogenic vascular scaffolds has attracted much interest since it not only preserves extracellular matrix (ECM) components but also imitates the geometry of the blood vessels and improves cellular attachment and proliferation; therefore, decellularized scaffolds could be suitable candidates for vascular transplantation. The decellularization of allogenic or xenogeneic vessels is an approach in which physical methods, enzymes, and detergents are recruited to remove the cellular components from the vascular scaffold [6, 7]. As a result, a biocompatible scaffold could be obtained that offers several advantages compared to the artificially synthesized 3D tissue scaffold, such as maintaining the natural ECM properties as well as biomechanical characteristics while lacking heterogenic antigens [5, 8–10]. Despite the fact that several studies have investigated various decellularization methods and provided valuable information regarding the characterization of acellular vessel scaffolds, there are ongoing debates over the most suitable decellularization strategy [5]. The decellularization process is followed by the recellularization procedure, in which the patient’s cells can be seeded into the acellular scaffold using in vivo acellular tissue transplantation or a bioreactor, leading to vascular tissue remodeling. However, a considerable number of the recellularized vessels lack proper cell repopulation and adequate endothelialization, which causes thrombosis after the transplantation [2, 11, 12]. Therefore, novel approaches should be used for efficient and uniform cell adhesion and migration within the scaffold. Improvement of recellularization is a challenging issue in the field of tissue engineering, and it may be achieved by modifications of the recellularization procedure, including the recruitment of the involved proteins in the attachment, migration, and differentiation of seeded cells in the scaffold [13].

Apelin is an endothelial cell-derived peptide, and its gene (*APLN*) encodes a 77-amino acid prepropeptide, which is cleaved to apelin-36, the mature form of the apelin peptide, or shorter forms including apelin-17, apelin-12, and apelin-13 [14]. Apelin promotes angiogenesis by the upregulation of vascular endothelial growth factor (VEGF), vascular endothelial growth factor receptor (VEGFR), and *hypoxia-inducible factor 1* (HIF-1), as well as activating PI3K/Akt/eNOS and AMPK/eNOS pathways [15, 16]. Moreover, it plays a role in the proliferation, survival, differentiation, and reduction of apoptosis in several cell lines, including endothelial, vascular smooth muscle, and mesenchymal stem cells [15, 17–19]. Several studies also revealed apelin’s participation in vascular regeneration [20, 21]. Masoud et al. reported that apelin expressed by the vascular graft is involved in vascular repair and prevents immune cell invasion of the graft as well as inflammation [21]. Therefore, it seems that using apelin might be a favorable approach for improving recellularization efficacy via the repopulation of acellular tissue, homogeneous endothelialization, reduction of inflammatory responses, and increase of the proliferation of attached cells within the scaffold. However, several problems, such as inhomogeneous distribution and degradation of apelin in the scaffold, might occur during the process of simple perfusion of the peptide. Immobilizing the peptide using covalent bonds to the scaffold might be a promising approach to address such issues [13, 22]. 1-ethyl-3-(3-dimethylaminopropyl) carbodiimide hydrochloride and N-hydroxysuccinimide (EDC-NHS) is a commonly used linker, which forms amide bonds between primary amine and carboxyl groups [23]. EDC, which is water soluble, interacts with carboxylic acids to produce reactive O-acylisourea intermediates, which subsequently form an amide bond through linkage to a nucleophile. To prevent the inactivation of EDC molecules and the cleavage of O-acylisourea intermediates by water oxygen atoms, the combination of EDC and sulfo-NHS can be used to create an active ester with carboxylic acid, which is stable, hydrophilic, and is slowly hydrolyzed in water. Ultimately, the sulfo-NHS ester is hydrolyzed quickly in the presence of amine nucleophiles, such as lysine side chains in peptide molecules, allowing for the creation of an amide bond [23]. Thus, EDC-NHS would be a promising linker for coupling carboxylic acid and amine groups of apelin-13 and ECM components of the acellular scaffold.

In the present study, we evaluated several decellularization protocols to determine the most efficient one and develop a modified recellularization process to enhance its efficacy. To improve the attachment, proliferation, viability, and retention of cells in the scaffold, the apelin-13 peptide was immobilized onto the vascular lumen scaffold using perfusion of the EDC-NHS linker prior to the cell perfusion.

## Materials and methods

### Materials

Fetal bovine serum (FBS) and penicillin/streptomycin were purchased from Bio-Idea (Tehran, Iran). Triton X-100 (TX) and sodium dodecyl sulfate (SDS) were procured from Merck (New Jersey, USA). 3-[(3-cholamidopropyl) dimethylammonio]-1-propanesulfonate (CHAPS) was obtained from Bio Basic (Markham, Canada). Peracetic acid was provided from NanoGenDarou (Semnan, Iran). Masson’s trichrome and orcein staining kit was obtained from Asia Pajoohesh (Mazandaran, Iran). The DNA extraction kit and the glycosaminoglycan (GAG) assay kit were from Pars Kia Gene (Kerman, Iran) and Kiazist (Hamedan, Iran), respectively. Apelin-13 enzyme-linked immunosorbent assay (ELISA) was purchased from Eastbiopharm (Zhejiang, China). EDC, NHS, Bio-Beads SM-2, and (3-(4,5-dimethylthiazol-2-yl)-2,5-diphenyltetrazolium bromide (MTT) were bought from Sigma-Aldrich (*Massachusetts, USA*). Human dermal fibroblasts, vascular smooth muscle cells (VSMCs), and human umbilical vein endothelial cells (HUVECs) were obtained from the Pasteur Institute of Iran (Tehran, Iran).

### Animals and harvesting the vessels

A total number of 80 adult male *Wistar* rats aged 4 months old were purchased from the animal house of Kerman University of Medical Sciences and used in this study for setting up the tests as well as performing assays. All animal procedures were approved by the Ethics Committee of Kerman University of Medical Sciences, Kerman, Iran (IR.KMU.AH.REC.1400.007) and performed in accordance with the Animal Research: Reporting of In Vivo Experiments (ARRIVE) guidelines of laboratory animal care. Animals were housed 2–3/cage in an animal room with a constant temperature (20–22 °C) and humidity (50–60%) under a 12-h interval light/dark cycle with free access to standard food. Next, the animals were anesthetized with an intraperitoneal injection of 100 mg/kg ketamine and 10 mg/kg xylazine before euthanasia, and subsequently, the aorta was harvested. The harvested vessels were then placed in the phosphate-buffered saline (PBS) medium supplemented with an antibiotic/antimycotic cocktail and kept at -20 °C overnight to facilitate the decellularization process.

### Decellularization protocols

**Fig. 1:**
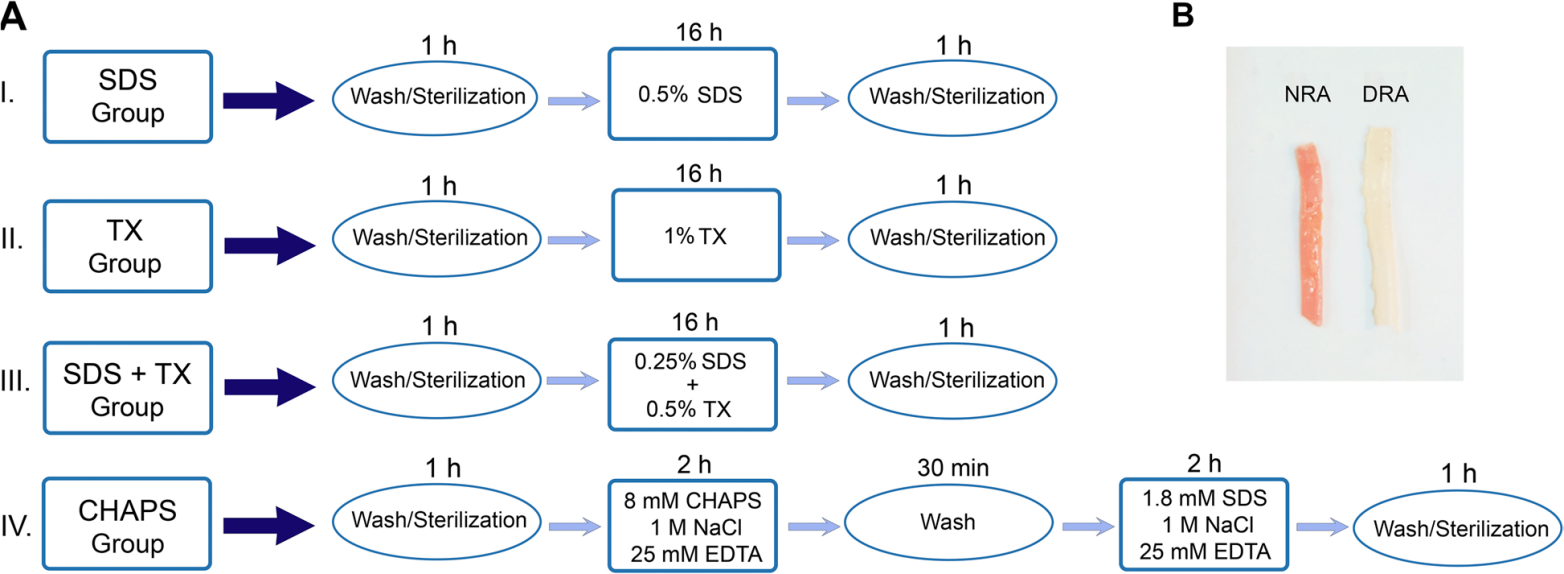
Schematic protocol indicates decellularization process using SDS, TX, and CHAPS detergents. Perfusion process was performed at room temperature and lasts 16 hours, while shaking process was performed at 37 °C (**A**) Gross view of NRA and DRA tissue decellularized using 0.25% SDS + 0.5% TX is shown in Fig. 1 indicating tissue transparency following the decellularization process (**B**). NRA: Native Rat Aorta, DRA: Decellularized Rat Aorta, SDS: Sodium Dodecyl Sulphate, TX: Triton X-100, CHAPS: 3-[(3-cholamidopropyl)

Previously published protocols used several decellularizing agents including detergents and enzymes such as TX, SDS, sodium deoxycholate (SDC), and CHAPS [24, 25]. In this study, the decellularization process with different combinations of detergents was carried out using a perfusion peristaltic pump (*Peristaltic Pump* P-1, Pharmacia Biotech, Sweden) at a 1.5 mL/min rate. Figure 1A depicts the decellularization protocols of the present study in detail. Briefly, vessels were decellularized by either detergent perfusion or shaking in the detergent solution. In the perfusion group, vessels were perfused with 50 mL of 0.5% SDS or 1% TX or a combination of 0.25% SDS and 0.5% TX for 16 h at room temperature. In the shaking group, the vessels were shaken at 37 °C in 50 mL of a detergent solution including 8 mM CHAPS, 1 M NaCl, and 25 mM EDTA for 2 h. Next, the tissues were washed by shaking in the PBS solution for 30 min, and then they were shaken at 37 °C in a solution of 1.8 mM SDS, 1 M NaCl, and 25 mM EDTA for 2 h. Afterward, the scaffolds were perfused with 0.1% peracetic acid for an hour and then rinsed with sterile PBS containing antibiotic/antimycotic. A cocktail was utilized to neutralize the acid and remove the residual detergent components from the vascular scaffold. The decellularized vessels were then stored in sterile PBS at 4 °C for further investigations including the evaluation of DNA, collagen, and GAG content as well as histological analysis. Moreover, all the experiments performed in the present study were carried out in three independent runs [13].

#### Histological analysis

To investigate the efficacy of the decellularization process, samples were fixed in 10% formalin solution for 24 h. The paraffin-embedded samples were stained with hematoxylin and eosin (H&E), Masson’s trichrome for distinguishing collagen, and orcein for the determination of elastic fibers according to the standard protocols before being examined under a light microscope (Olympus BX53, Japan).

#### DNA quantification

To evaluate the quality of decellularization, the total DNA content of native and decellularized vessels was measured. Firstly, the samples were homogenized in the lysis buffer provided in the DNA extraction kit. Total DNA content was extracted according to the manufacturer’s instructions, and the DNA concentration was quantified using a spectrophotometer (ND-1000, Thermo Fisher Scientific, USA) at the 260 nm absorbance wavelength and was normalized according to the wet tissue weight. The results are expressed as ng/mg wet weight of samples.

#### Hydroxyproline quantification

A colorimetric assay was employed to assess the collagen content of native and decellularized samples through the measurement of hydroxyproline [26]. Concisely, homogenized samples were incubated with cupric sulfate, sodium hydroxide, and hydrogen peroxide at 80 °C for 5 min and then were cooled. Afterward, sulfuric acid and ρ-dimethylaminobenzaldehyde in 1-propanol were added to the samples, which were then incubated at 80 °C for 30 min, and absorption was read at 560 nm using a spectrophotometer (Epoch, BioTek, USA). Moreover, the hydroxyproline level was directly determined using a hydroxyproline standard curve and normalized using wet tissue weight.

#### GAG quantification

Native and decellularized samples were first digested in papain overnight at 65 °C to assess the GAG content using the GAG assay kit (Kiazist, Iran). Then, the samples were centrifuged (Sigma 3-16PK, Germany) at 8000 g for 15 min, the protein precipitant solution was added to the supernatant, and the samples were centrifuged again at 8000 g for 15 min. Subsequently, a GAG reagent was added to the samples and total absorption was measured at 560 nm using a spectrophotometer (Epoch, BioTek, USA). Moreover, the chondroitin sulfate standard curve was used to directly determine the GAG content and the total tissue GAG content was normalized using wet tissue weight.

#### Field emission scanning electron microscopy (FESEM)

To characterize the topography of the luminal surface, native and decellularized tissues were freeze-dried (Eleya, Japan), mounted on aluminum stubs, and coated with a thin layer of gold. The analysis of morphology and sample structure was performed at a voltage of 15 kV by FESEM (TESCAN MIRA3, *TESCAN, the Czech Republic*) [27].

### Immobilization of apelin-13 on the scaffold

In the present study, apelin-13 was attached to the scaffold via passive and active absorption to investigate the efficacy of EDC-NHS linkers in the attachment of apelin-13. Decellularized vessels were perfused with 6 μg/mL apelin-13 for 4 h at a rate of 0.5 mL/min for passive absorption. However, in the EDC-NHS conjugated group, the vascular scaffold was first perfused with 4.8 mg/mL EDC and 12 mg/mL NHS in PBS for 1 h and then perfused with 6 μg/mL apelin-13 for 4 h at a rate of 0.5 mL/min. The efficacy of apelin-13 attachment to the scaffold was assessed using an apelin-13 ELISA kit. Moreover, no apelin-13 treatment was performed for the vascular scaffold in the negative control group. The group with higher apelin-13 attachment efficacy was selected for further studies.

### Viability and proliferation of cells in the decellularized scaffold

The viability of cells in acellular scaffolds was assessed using two different washing approaches. In the first approach, the viability of seeded cells in the presence of acellular scaffolds was assessed by the MTT assay according to the manufacturer’s instructions after washing them with the PBS solution for 48 h without Bio-Beads SM-2 incubation. In brief, decellularized scaffolds (with and without apelin-13) were placed in 12-well tissue culture plates and cells were seeded at a density of 2.5 × 10^5^ cells/well for 1, 3, and 7 days.

In the second approach, the MTT assay was performed to evaluate the viability and proliferation of seeded cells in the acellular scaffolds incubated with 1 g/mL (dry weight) Bio-Beads SM-2 for 48 h as described in the previous section using the same cell density. At the end of the 1st, 3rd, and 7th days, the MTT solution was added to the wells and the cells were incubated at 37 °C for 3 h in the dark (cell culture incubator, Jal Teb, Iran). Finally, the absorbance of the produced color was evaluated at 570 nm using a spectrophotometer (Epoch, BioTek, USA). Higher color production indicates a higher number of viable cells.

### Recellularization of acellular vascular scaffold

In the present study, the recellularization process was performed following the sterilization of the vascular scaffold using an antibiotic/antimycotic cocktail. Afterward, decellularized scaffolds (with and without apelin-13) were rinsed with PBS. The recellularization process was carried out on three consecutive days by the perfusion of 1.5 million cells/mL human fibroblast cells, HUVECs, and VSMCs at 37 °C and a perfusion rate of 0.5 mL/min using a peristaltic pump. Next, the efficacy of the recellularization process was evaluated 7 days after the end of the procedure via histological, fluorescent (DP71, Olympus fluorescent microscope, Japan), and FESEM analyses as well as the measurement of the DNA content.

### Functional study

To assess the mechanical properties and uniaxial tensile strength of decellularized and recellularized vessels compared to the native ones, a Testometric tensile testing machine (Testometric Machine M350-10CT, Rochdale, UK) was used, and tests were performed at room temperature. Due to variant aorta wall thickness, the results were adjusted according to the tension per unit thickness of the specimen to avoid the effect of aorta wall thickness on the calculation of tensile strength. The calculation of tension was based on the formula T = F/I in which T denotes tension in N/mm, F denotes the acquired force in N, and I indicates the specimen width in mm.

### Statistical analysis

One-way analysis of variance (ANOVA) and TUKEY post hoc tests were performed to compare groups using GraphPad Prism software version 8 for Windows (GraphPad Software Inc., San Diego, CA, USA), and *P*-values less than 0.05 were considered statistically significant.

## Results

### Characteristics of native and decellularized rat aorta

#### Quantification of DNA, hydroxyproline, and GAG content

**Fig. 2:**
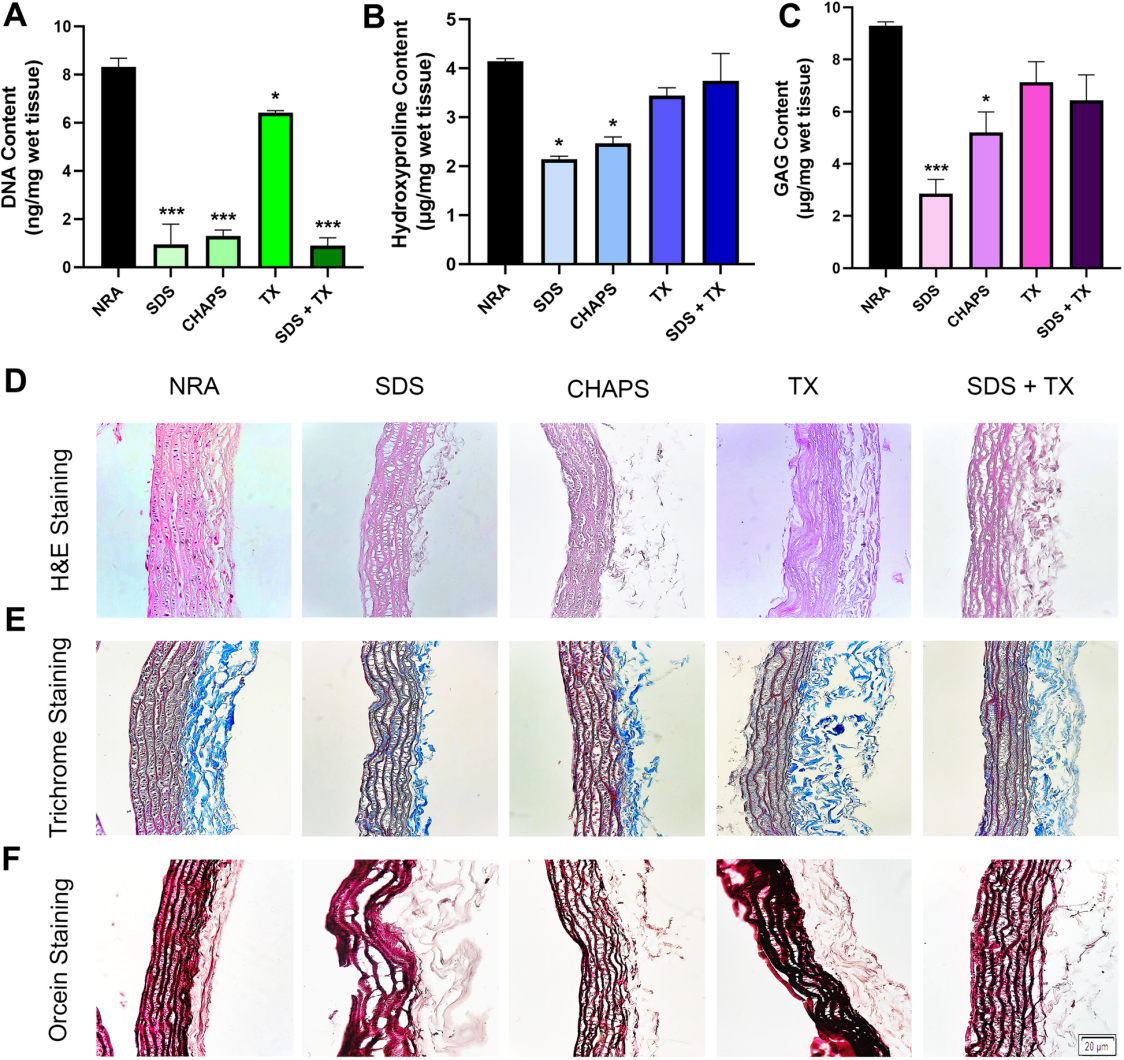
Biochemical and histological analysis of NRA and acellular tissues decellularized using 0.5% SDS, CHAPS, 1% TX, and combination of 0.25% SDS + 0.5% TX is shown in Fig. 2. Assessment of DNA content reveals that decellularization of tissues using 0.25% SDS + 0.5% TX eliminates cell components from native tissue to a greater extent compared to other detergents (**A**), Hydroxyproline content is preserved in tissues decellularized using 1% TX and 0.25% SDS + 0.5% TX (**B**), GAG content does not decrease significantly in tissues decellularized using 1% TX and 0.25% SDS + 0.5% TX, which suggests efficacy of these protocols in preserving ECM (**C**), H&E staining of native and acellular vessels (**D**), Masson’s trichrome staining (**E**), and orcein staining (**F**) of NRA and DRA are also shown in this Figure suggesting removal of cell components in 0.25% SDS + 0.5% TX group with little damage to ECM proteins.(* difference with NRA, *P* < 0.05; and *** difference with NRA, *P* < 0.001). NRA: Native Rat Aorta, SDS: Sodium Dodecyl Sulphate, CHAPS: 3-[(3-cholamidopropyl) dimethylammonio]-1-propanesulfonate, TX: Triton X-100, SDS + TX: Sodium Dodecyl Sulphate + Triton X-100

The efficacy of the decellularization process is determined by assessing the elimination of cellular content as well as the preservation of ECM components including proteins and GAG. In the present study, measuring the DNA content indicated the efficiency of cell removal from the host tissue and the results revealed that the DNA content in all decellularized groups was significantly reduced; however, using the combination of 0.25% SDS and 0.5% TX detergents is a more promising protocol for removing the cellular content (0.9 ± 0.32 ng/mg wet tissue in the SDS + TX group vs. 8.32 ± 0.35 ng/mg wet tissue in the native rat aorta (NRA), *P* < 0.001) (Fig. 2A). Moreover, it has been shown that the DNA content in tissues decellularized using 1% TX is significantly higher than that in the other acellular groups. The hydroxyproline content of the tissue, which is an indicator of the collagen level, declined significantly in the tissues decellularized using 0.5% SDS and CHAPS compared to the native tissue (2.14 ± 0.06 μg in the SDS and 2.47 ± 0.13 μg in the CHAPS groups vs. 4.145 ± 0.05 μg in NRA per mg of wet tissue weight, *P* < 0.05); however, the hydroxyproline content of 1% TX and 0.25% SDS + 0.5% TX groups did not significantly decrease compared to the NRA (Fig. 2B). Similarly, the quantification of the GAG content in native and acellular tissues demonstrated a significant decrease in the GAG level of tissues decellularized using 0.5% SDS and CHAPS (2.85 ± 0.055 μg/mg wet tissue weight (*P* < 0.001) and 2.47 ± 0.78 μg/mg wet tissue weight (*P* < 0.05), respectively) compared to the native control (9.29 ± 0.14 μg/mg wet tissue weight); however, the GAG content in other acellular tissues including the 1% TX and 0.25% SDS + 0.5% TX groups did not show a significant difference compared to NRA (Fig. 2C).

#### Histology and FESEM analysis

**Fig. 3:**
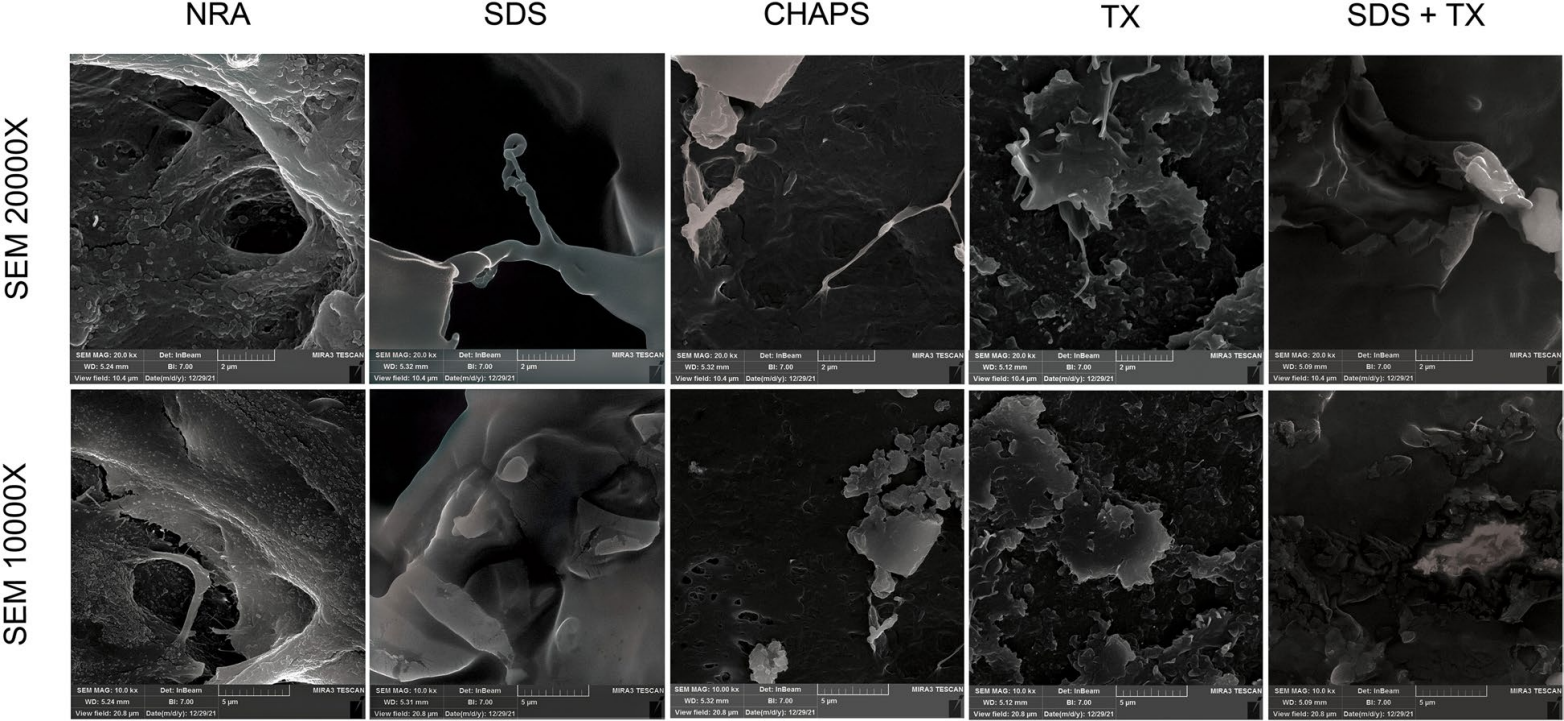
SEM analysis of the luminal side of NRA and DRA confirms the elimination of cell components in tissues decellularized using 0.5% SDS and 0.25% SDS + 0.5% TX. Cellular remnants are observed as white spots in 1% TX group and to a lower extent in CHAPS group. NRA: Native Rat Aorta, DRA: Decellularized Rat Aorta, SDS: Sodium Dodecyl Sulphate, CHAPS: 3-[(3-cholamidopropyl) dimethylammonio]-1-propanesulfonate, TX: Triton X-100, SDS + TX: Sodium Dodecyl Sulphate + Triton X-100

The gross view of all decellularized tissues using different detergents was similar in terms of color and transparency, but tissues decellularized using perfusion with 0.5% SDS lacked consistency compared to other decellularized tissues. The comparison of native and acellular aorta scaffolds decellularized using a combination of 0.25% SDS and 0.5% TX is shown in Fig. 1B, which indicates the transparency of vascular tissue after the decellularization process. H&E staining revealed the elimination of cell components in acellular scaffolds compared to the native ones. In addition, vascular tissue demolition was observed in the 0.5% SDS group, while tissue structure was consistent in other acellular groups and did not change considerably compared to the native one (Fig. 2D). The grading system of Masson’s trichrome and orcein stains used in this study was 1+, 2+, 3+, and 4+ for tissues stained 0–25%, 26–50%, 51–75%, and 76–100%, respectively. Moreover, Masson’s trichrome staining depicted the presence of collagen fibers at the media and adventitia layers, which demonstrated a reduction of collagen fibers in 0.5% SDS and CHAPS groups. The semi-quantitative analysis indicated the grading of + 2 for NRA, 1% TX, and 0.25% SDS + 0.5% TX groups, and + 1 for 0.5% SDS and CHAPS groups, which suggests the considerable decline of the collagen content in the 0.5% SDS group (Fig. 2E). In addition, orcein staining revealed the preservation of elastin fibers in the media in CHAPS, 1% TX, and 0.25% SDS + 0.5% TX groups, which were shown to have a grading of + 4, while the elastin content in the 0.5% SDS group had a grading of + 3. Additionally, the elastin-specific staining suggests the significant destruction of elastic fibers in 0.5% SDS decellularized tissue (Fig. 2F). The FESEM analysis confirmed the reduction or absence of nuclear components and endothelial cells in the three-dimensional structure of aorta lumen in vessels decellularized using 0.5% SDS and 0.25% SDS + 0.5% TX, while the remnants of cell components were observed in the 1% TX group, and to a lower extent, in the CHAPS group as white spots. Moreover, FESEM imaging indicated damage to vascular tissue decellularized using 0.5% SDS, while other acellular tissues were not affected by the decellularization process. Furthermore, the FESEM analysis confirmed the histological analysis and demonstrated the removal of cell components in decellularized vessels compared to native tissue as shown in Fig. 3. According to the obtained results, vessels decellularized using 0.25% SDS and 0.5% TX were selected for performing other investigations including the assessment of peptide attachment and recellularization.

### Apelin-13 immobilization in the acellular vascular scaffold

**Fig. 4:**
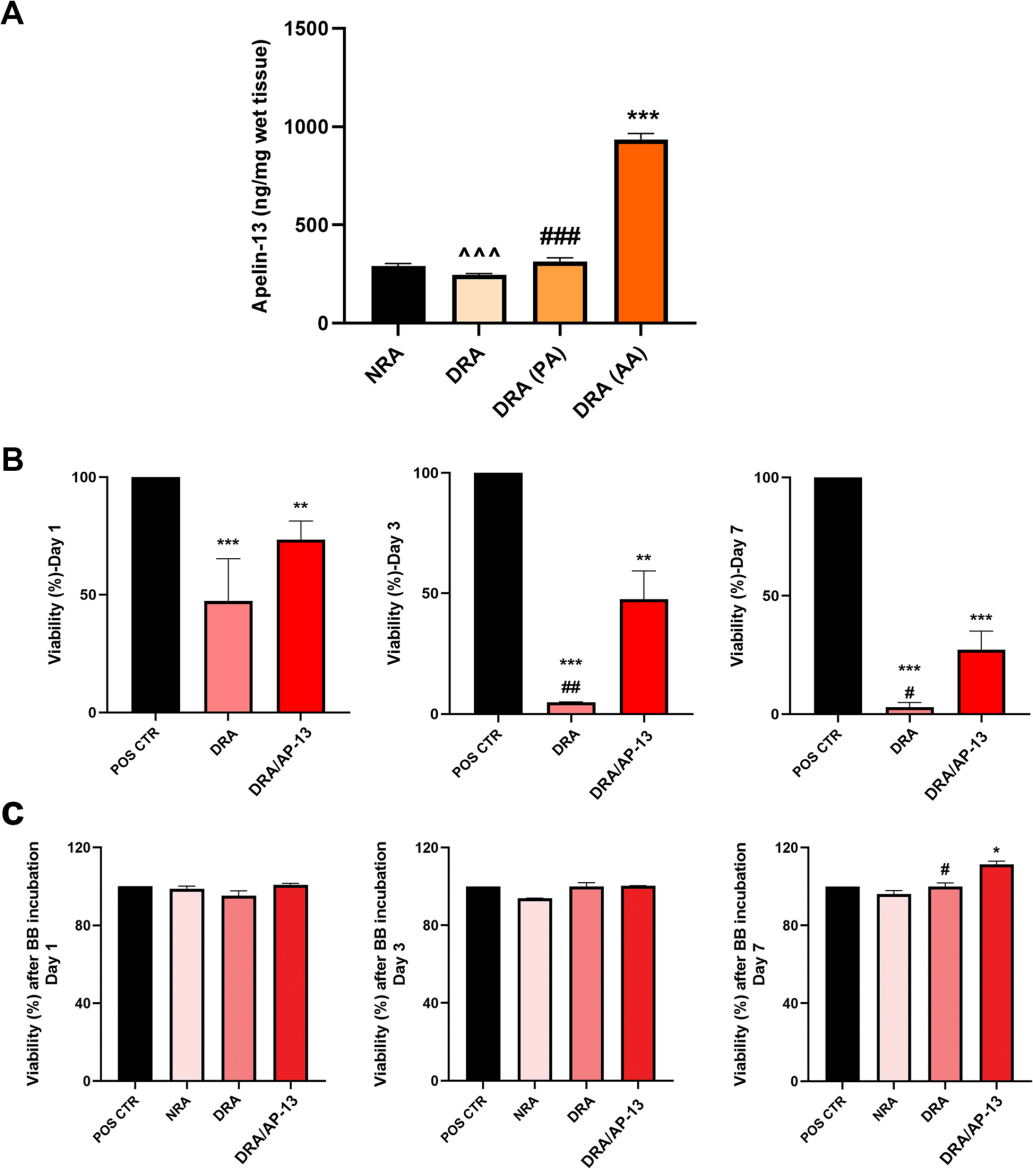
Comparison of apelin-13 attachment to acellular scaffolds via passive (PA) and active absorption (AA) indicates higher peptide attachment after bioconjugation of apelin-13 to the scaffold using EDC-NHS linker (**A**). (*** difference with NRA, *P* < 0.001; ### difference with DRA (AA), *P* < 0.001; and ^^^ difference with DRA (PA), *P* < 0.001). Cell viability within acellular tissues was found to be significantly lower in decellularized tissues washed by PBS compared to the control group, which is possibly due to toxic effects of detergent remnants on cell survival (**B**) The results of the incubation of the acellular scaffold with Bio-Beads SM-2 indicated no significant reduction in cell viability in this group. Cell proliferation was also higher in acellular tissues bioconjugated with apelin-13 compared to non-conjugated (**C**) (* difference with POS CTR, *P* < 0.05; ** difference with POS CTR, *P* < 0.01; *** difference with POS CTR, *P* < 0.001; # difference with RRA/AP-13, *P* < 0.05, and ## difference with RRA/AP-13, *P* < 0.01). NRA: Native Rat Aorta, DRA: Decellularized Rat Aorta, PA: Apelin-13 Passive Absorption, AA: Apelim-13 Active Absorption, POS CTR: Positive control, RRA: Recellularized Rat Aorta, RRA/AP-13: Recellularized Rat Aorta Conjugated with Apelin-13

To evaluate the difference between the amounts of apelin-13 attached to the acellular scaffold using passive and active absorption techniques, ELISA was performed to measure the apelin-13 level. The results showed that our active absorption protocol using the EDC-NHS linker considerably increased the amount of apelin-13 attached to the scaffold (935.38 ± 30.17 μg/mg wet tissue weight, *P* < 0.001) compared to the passive absorption (314.25 ± 19.11 μg/mg wet tissue weight), which indicates the higher efficiency of using these linkers for peptide attachment purposes (Fig. 4A). Therefore, the decellularized tissue bioconjugated with apelin-13 using EDC-NHS was selected for further studies.

### Cell viability in acellular scaffolds in the presence and absence of apelin-13

According to our results, washing the acellular scaffold with PBS left detergent remnants in the scaffold, which negatively affect cell survival. Cell viability within acellular tissues was found to be significantly lower in decellularized tissues washed by PBS compared to the control group; cell viability on the 1st, 3rd, and 7th days was 73.37% ± 7.99, 47.6% ± 11.69, and 27.3% ± 7.89%, respectively, for decellularized rat aorta conjugated with apelin-13 (DRA/AP-13 group), whereas it was 47.34% ± 17.98, 4.39% ± 0.13, and 2.95% ± 2%, respectively, for decellularized rat aorta (DRA group). However, cell viability was considerably lower in the non-conjugated scaffold compared to the acellular tissue conjugated with apelin-13 on days 3 and 7 (Fig. 4B). The results of the incubation of the acellular scaffold with Bio-Beads SM-2 indicated no significant reduction in cell viability in this group; cell viability on the 1st, 3rd, and 7th days was 100.79% ± 0.7, 100.34% ± 0.08, and 111.24% ± 1.7% for the DRA/AP-13 group incubated with Bio-Beads SM-2, respectively, whereas it was 95.14% ± 2.54, 100.03% ± 1.89, and 100.04% ± 1.76% for the DRA group with Bio-Beads SM-2, respectively. Moreover, it was shown that apelin-13 can significantly stimulate cell proliferation after 7 days in scaffolds incubated with Bio-Beads SM-2 (*P* < 0.05) (Fig. 4C).

### Characteristics of recellularized rat aorta (RRA)

#### Assessment of DNA, hydroxyproline, and GAG content

**Fig. 5:**
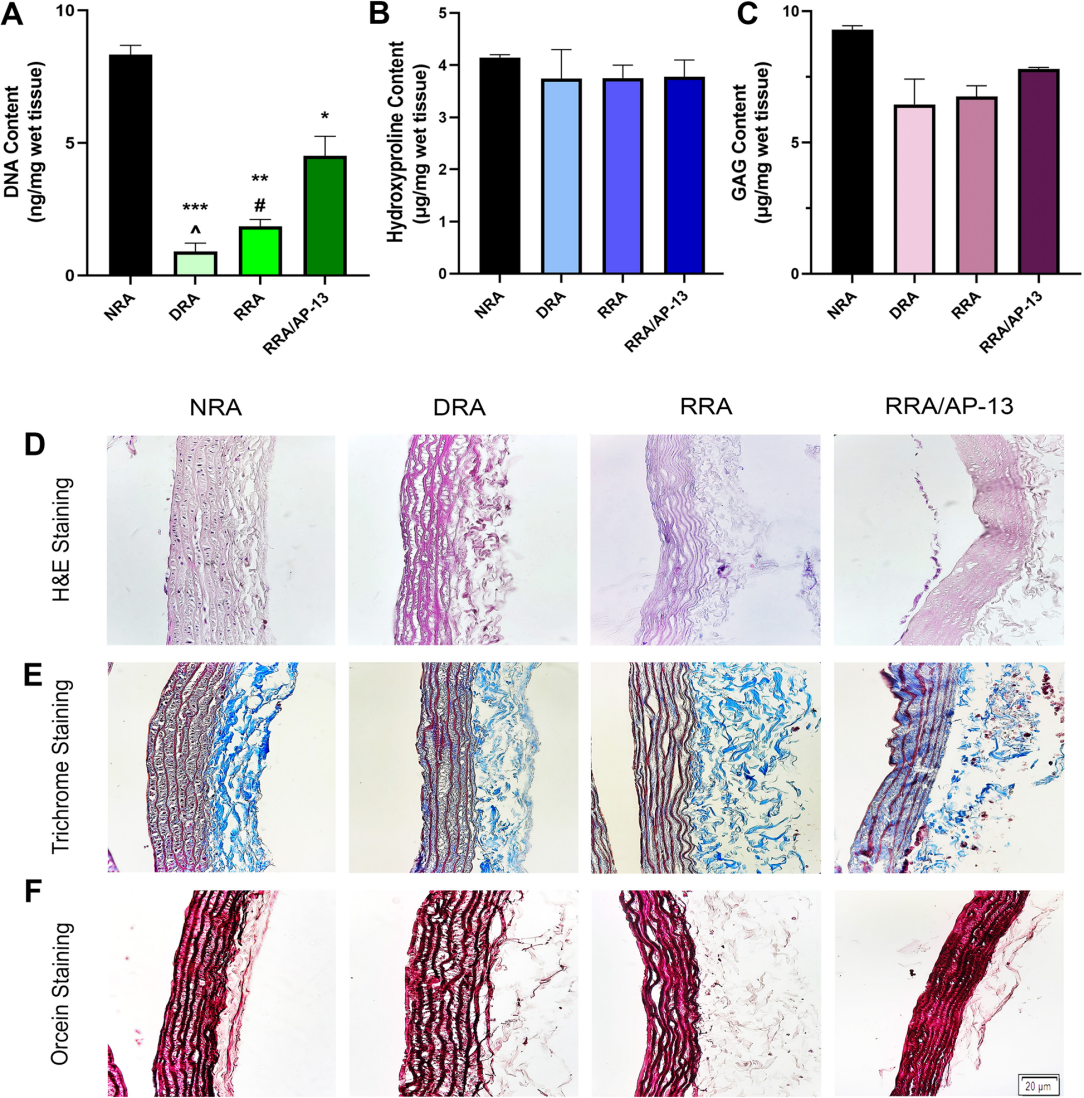
Biochemical and histological analysis of NRA, DRA, RRA, and RRA/AP-13 tissues. The DNA content of the RRA group was significantly higher than that of the DRA group, while it was significantly lower than recellularized tissue bioconjugated with apelin-13 (**A**), Hydroxyproline content of all recellularized tissues does not show a considerable decrease compared to DRA or NRA groups (**B**), and GAG content in recellularized tissues does not change significantly compared to native and acellular tissues (**C**), H&E staining indicates attachment of endothelial cells to the luminal surface of vessel (**D**), Masson’s trichrome staining (**E**), and orcein staining (**F**) of NRA, DRA, RRA, and RRA/AP-13 groups are shown in Fig. 6 representing consistent structure of collagen and elastin fibers in the vascular tissue (* difference with NRA, *P* < 0.05; ** difference with NRA, *P* < 0.01; *** difference with NRA, *P* < 0.001; # difference with RRA/AP-13, *P* < 0.05; and ^ difference with RRA, *P* < 0.05). NRA: Native Rat Aorta, DRA: Decellularized Rat Aorta, RRA: Recellularized Rat Aorta, RRA/AP-13: Recellularized Rat Aorta Conjugated with Apelin-13

To assess the recellularization outcome, the DNA content was measured and the results indicated a significant increase in the DNA content of the RRA/AP-13 group (4.51 ± 0.74 ng/mg wet tissue) compared to the RRA (1.85 ± 0.25 ng/mg wet tissue) and DRA (0.9 ± 0.3 ng/mg wet tissue) groups (*P* < 0.01 and 0.001, respectively). Furthermore, the DNA content of the RRA group was significantly higher than that of the DRA group (Fig. 5A). Regarding the ECM content of recellularized tissues, the hydroxyproline content of RRA and RRA/AP-13 groups did not change significantly compared to the DRA and NRA groups (3.75 ± 0.25 μg/mg wet tissue and 3.77 ± 0.32 μg/mg wet tissue vs. 3.74 ± 0.056 μg/mg wet tissue and 4.14 ± 0.05 μg/mg wet tissue, respectively, *P* > 0.05). Furthermore, the GAG content of the RRA and RRA/AP-13 groups did not show a significant increase compared to the DRA group (*P* > 0.05) (Fig. 5B and C, respectively).

#### Histological, fluorescent, and FESEM analyses

**Fig. 6:**
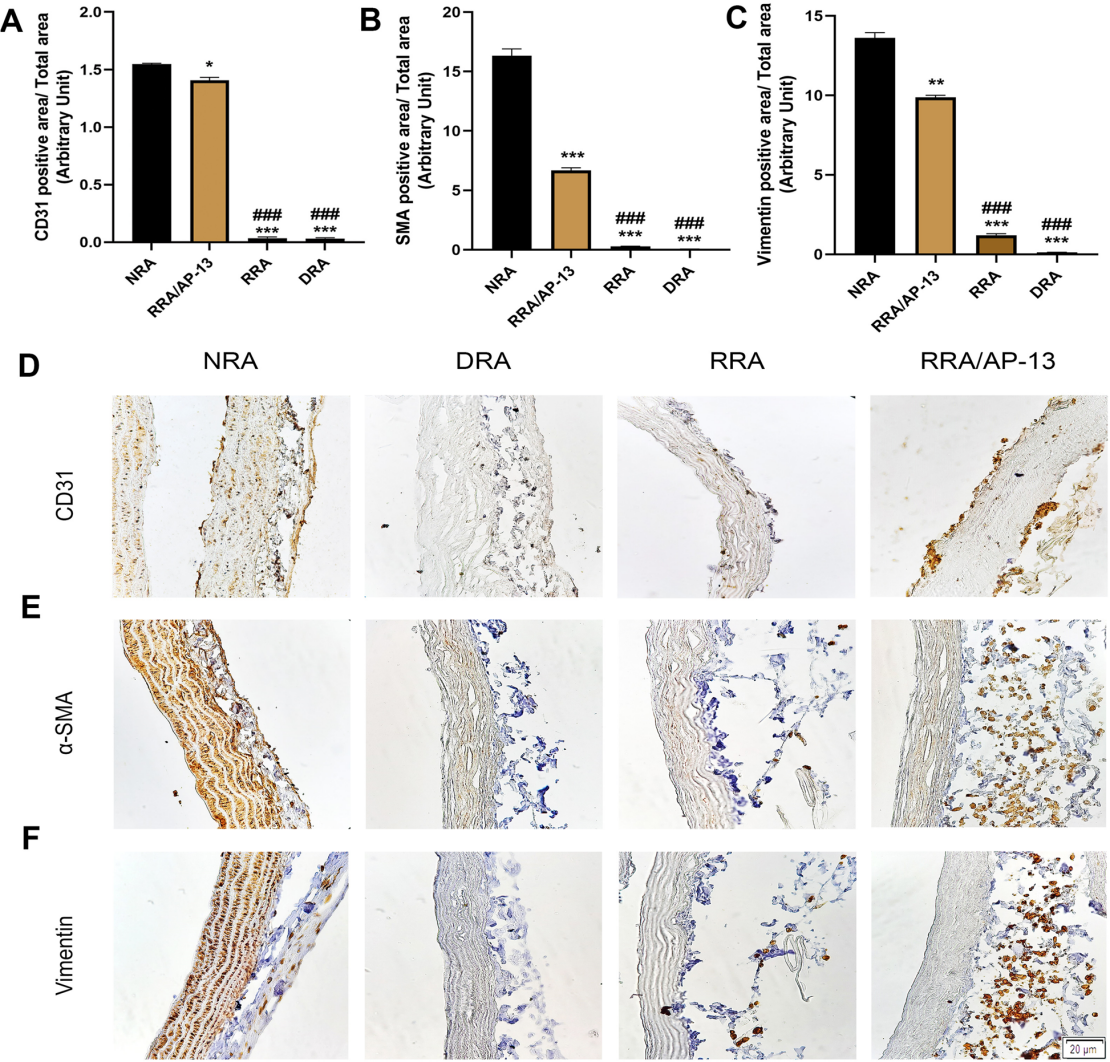
Quantitative analysis of CD31 (**A**), α-SMA (**B**), and vimentin (**C**) markers are represented in Fig. 6 indicating significant attachment of endothelial, smooth muscle cell, and fibroblast cells to RRA-AP-13 group compared to RRA and DRA groups. Moreover, IHC staining of CD31 (D), α-SMA (**E**), and vimentin (**F**) markers are shown in Fig. 6. Brown spots show presence of endothelial, vascular smooth muscle cells, and fibroblast cells in CD31, α-SMA, and vimentin staining, respectively (* difference with NRA, *P* < 0.05; ** difference with NRA, *P* < 0.01; *** difference with NRA, *P* < 0.001; ### difference with RRA/AP-13, *P* < 0.001). NRA: Native Rat Aorta, DRA: Decellularized Rat Aorta, RRA: Recellularized Rat Aorta, RRA/AP-13: Recellularized Rat Aorta Conjugated with Apelin-13

**Fig. 7:**
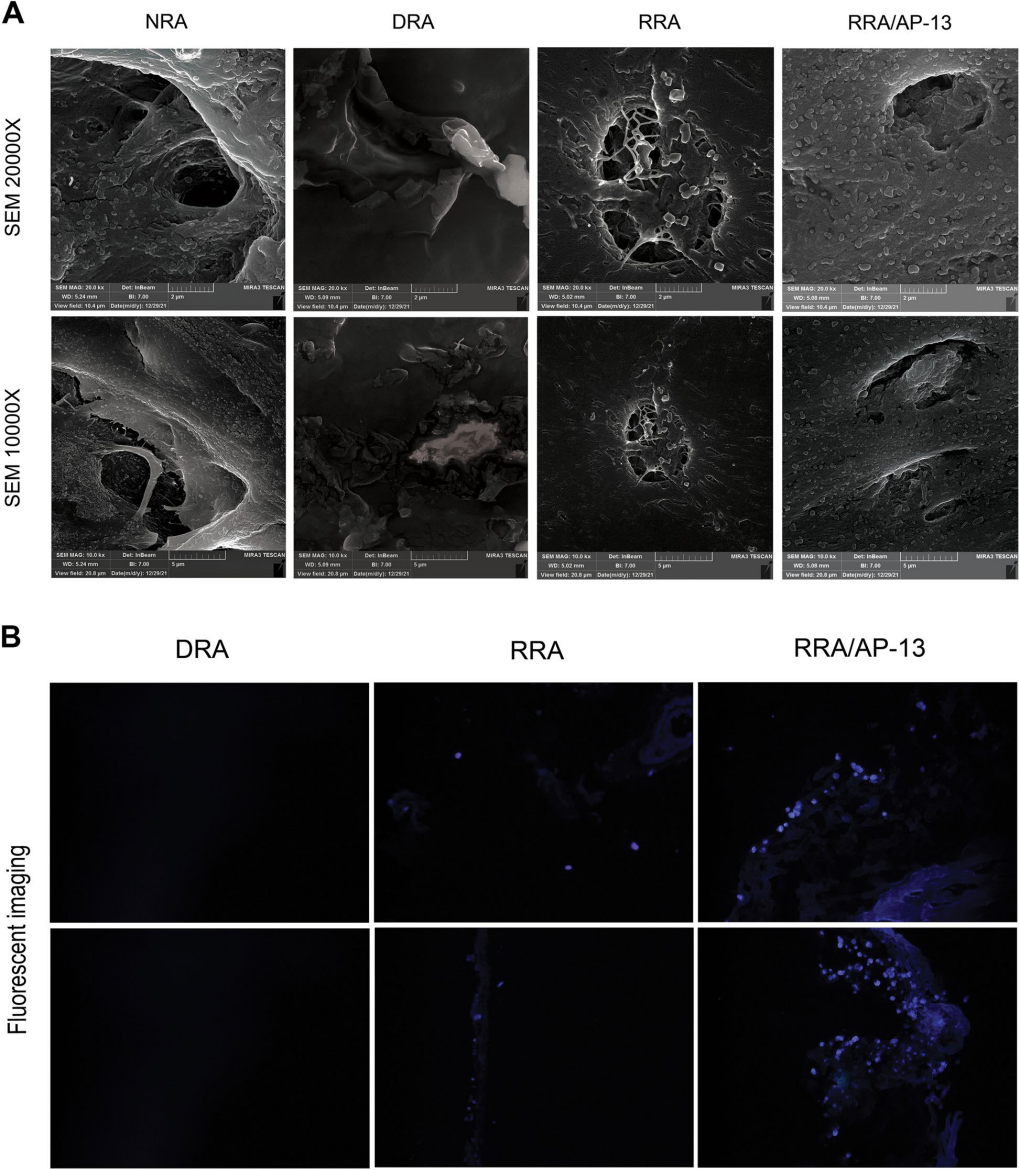
FESEM analysis of the luminal side of NRA, DRA, RRA, and RRA/AP-13 demonstrates uniform repopulation of acellular scaffold after the recellularization process and higher cell attachment to RRA/AP-13 tissue compared to RRA (**A**). The presence of a higher number of cells stained by Hoechst 33342 stain in RRA/AP-13 tissue demonstrates increased efficacy of cell attachment to RRA/AP-13 scaffold compared to the RRA tissue. The first and second rows represent different sides of tissue (**B**). NRA: Native Rat Aorta, DRA: Decellularized Rat Aorta, RRA: Recellularized Rat Aorta, RRA/AP-13: Recellularized Rat Aorta Conjugated with Apelin-13

The histological analysis indicated the presence of endothelial cells in the luminal side of the vessel in the RRA/AP-13 group. However, no cells were observed in the RRA group, which might be due to the limited number of cells seeded in the acellular tissue (Fig. 5D). Moreover, the quantitative analysis of immunohistochemical (IHC) staining for CD31, α-SMA, and vimentin markers suggested considerable elevation in the number of endothelial cells, smooth muscle cells, and fibroblasts in the RRA/AP-13 group (1.4% ± 0.02, 6.66% ± 0.23, and 9.87% ± 0.13%, respectively) compared to RRA (0.03% ± 0.01, 0.28% ± 0.01, and 1.2% ± 0.09%, respectively) and DRA groups (0.03% ± 0.007, 0.05% ± 0.01, and 0.13% ± 0.005%, respectively). Brown spots show the presence of endothelial, vascular smooth muscle, and fibroblast cells in CD31, α-SMA, and vimentin stainings, respectively (Fig. 6). The semi-quantitative analysis of Masson’s trichrome and orcein staining also revealed the grades of + 2 and + 4 in all NRA, DRA, RRA, and RRA/AP-13 groups (Fig. 5E and F). The FESEM image confirmed the presence of attached cells in the three-dimensional structure of the rat aorta. Additionally, a more comprehensive analysis of the luminal surface of recellularized tissues using FESEM studies revealed the presence of cells on the luminal side of both RRA and RRA/AP-13 groups; however, the number of attached cells in the RRA/AP-13 group was considerably higher than that in the RRA group and they covered the luminal surface uniformly (Fig. 7A). Consistent with the other results, the fluorescent staining of recellularized tissues also confirmed the repopulation of acellular scaffolds in both recellularized groups; however, the number of attached cells, as well as their uniformity, in the RRA/AP-13 group was considerably higher than in the RRA group (Fig. 7B).

#### Mechanical properties of acellular and recellularized rat aorta

**Fig. 8:**
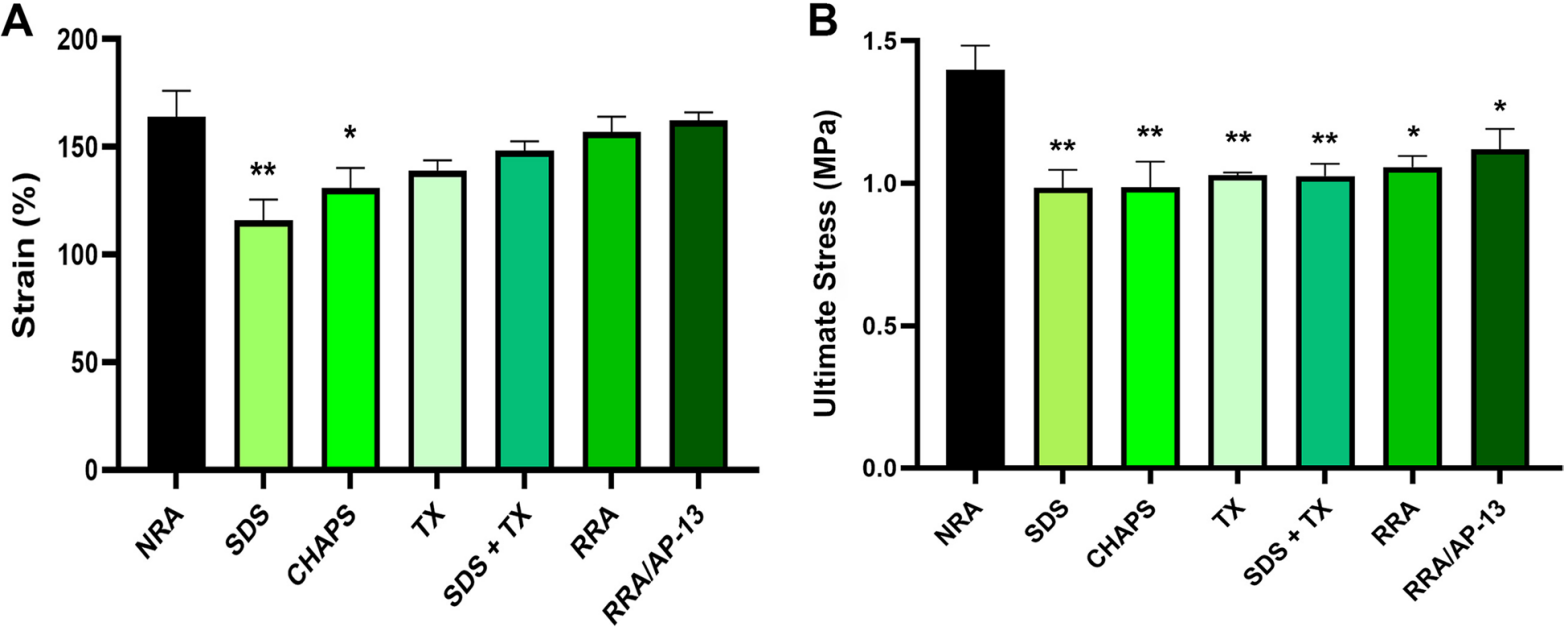
Mechanical testing of native, decellularized, and recellularized vascular tissues. Tissues elongation until the rupture is indicated as strain. Our results exhibited a reduction in the maximum strain to rupture of 0.5% SDS and CHAPS groups compared to that of native tissues (116% ± 6.79, 139.1% ± 3.24, and 164% ± 8.54%, respectively) (**A**). maximum force tolerated by tissues before rupture is presented as ultimate stress. Ultimate stress tolerated before rupture was significantly lower in both RRA and RRA/AP-13 groups compared to that of NRA (**B**) (* difference with NRA, *P* < 0.05; and ** difference with NRA, *P* < 0.01). NRA: Native Rat Aorta, SDS: Sodium Dodecyl Sulphate, CHAPS: 3-[(3-cholamidopropyl) dimethylammonio]-1-propanesulfonate, TX: Triton X-100, SDS + TX: Sodium Dodecyl Sulphate + Triton X-100, RRA: Recellularized Rat Aorta, RRA/AP-13: Recellularized Rat Aorta

Maintenance of mechanical properties in decellularized and recellularized tissues has great importance in determining vascular functionality and long-term success after vascular transplantation. Regarding decellularized tissues, our results exhibited a reduction in the maximum strain to rupture of 0.5% SDS and CHAPS groups compared to that of native tissues (116% ± 6.79, 139.1% ± 3.24, and 164% ± 8.54%, respectively), while ultimate stress was decreased in all decellularized tissues (*P* < 0.01). Moreover, ultimate stress and strain to rupture levels were significantly lower in both RRA and RRA/AP-13 groups compared to the NRA group (1.05 ± 0.02 MPa, 1.12 ± 0.05 MPa, and 1.4 ± 0.05 MPa, respectively, *P* < 0.05) (Fig. 8).

## Discussion

Recellularization of the acellular vessel scaffold is a promising approach for the treatment of patients who are candidates for vascular graft surgery. To generate a functional vascular graft, the developed vessel must meet two crucial criteria: 1) A proper scaffold should be utilized; 2) an optimized cellularization method should be used [28]. The three-dimensional acellular vascular scaffold is made up of an ECM, which consists of collagen, elastin, and GAG. The degradation of vascular matrix proteins affects the remodeling of the vascular wall matrix, which can cause stiffness, leakage, and rupture of the vascular wall. Moreover, the breakdown of vascular matrix proteins leads to proliferation and neointima formation within the vessel wall and makes blood vessels susceptible to uncontrolled enlargement during diastole. Furthermore, vascular ECM consists of fibrous proteins such as elastin and collagen, proteoglycans, and GAGs, and their interaction with cells regulates cell proliferation, adhesion, and migration [29]. Elastin serves as more than only a structural protein in arterial walls for the storage of energy, and its degradation increases arterial wall stiffness. Collagen is another stiff protein that not only restricts vessel distension but also takes part in cell adhesion, migration, differentiation, and proliferation. Other proteins include fibronectin, which alters the elastic modulus and mean stress of the vessel wall, and fibulin, which interacts with ECM proteins, such as elastin, and its shortage causes several problems including an aneurysm, rupture, and hemorrhage [29]. Thus, the preservation of ECM components during decellularization is of great importance. The presence of collagen and elastin fibers increases the tensile strength needed for the tolerance of pulsatile blood pressure, and GAG creates the viscoelastic characteristics of the vessels [30]. The results of the present study indicate that using the 0.25% SDS + 0.5% TX combination is the most efficient method for the elimination of cells as well as the preservation of ECM components including the collagen and GAG content in the native tissue. The aforementioned detergents play their role in cell removal through cell degradation and releasing cell content to the environment. Detergents such as SDS and SDC degrade cells by dissociating proteins from the cell membrane, while TX functions by breaking lipid-lipid and lipid-protein interactions [31–33]. Moreover, the histological analysis revealed a loss of tissue cohesion in case of using SDS as the decellularizing agent, while tissue cohesion underwent the least changes by using TX. These findings are consistent with those of previous studies [9, 25, 34]. Crapo and White indicated that certain detergents, namely, SDS and SDC, degrade the protein structure completely, while non-ionic detergents such as TX maintain the proteins in their intact form, which explains the reason for tissue destruction in the presence of SDS [9, 34]. Additionally, in our study, collagen and GAG levels were significantly decreased in tissues decellularized using SDS and CHAPS compared to the native ones, and these findings confirm the results of previous studies to some extent [5, 25]. Thus, using a combination of SDS and TX would be a promising approach toward obtaining a suitable decellularized scaffold, which not only removes the majority of tissue cells but also maintains the natural tissue structure.

The decellularization process may also alter the biochemical and biomechanical characteristics of native vessels. According to the findings of the present study, vessels decellularized using SDS and CHAPS detergents show a considerable decrease in the maximum strain to the rupture level. In addition, ultimate stress was significantly reduced in all acellular tissues compared to the native ones. It is suggested that vascular elastic fibers are responsible for the reversible vascular structure when the heart pumps blood. On the other hand, the presence of collagen stiffens the vascular structure and strengthens the vessels against high blood pressure [35]. Thus, collagen fibers enable vessels to withstand high blood pressure as their stiffness prevents vascular rupture. It is assumed that the decreased strength of arteries in SDS and CHAPS groups is due to the reduction of collagen levels after the decellularization process. Therefore, the remaining proteins and GAGs contribute to cell proliferation, adhesion, and migration within the scaffold and improve the efficacy of recellularization. Moreover, the preservation of ECM proteins, such as elastin, prevents vascular aneurysms, ruptures, and hemorrhages.

Furthermore, in this study, apelin-13 was immobilized on the vascular acellular scaffold using an EDC-NHS linker, which results in elevated cell attachment to the acellular scaffold. The EDC-NHS linker functions by forming zero-length cross-linking bonds between the carboxyl group of glutamic or aspartic acid and free amino groups of other polypeptides such as apelin-13 [36, 37]. Considering the role of apelin-13 in elevating the number of cells attached to the scaffold as well as increasing viability, proliferation, and cell migration, the attachment of this peptide to the acellular scaffold using the EDC-NHS linker enhances its accessibility and concentration for improving cell viability, proliferation, attachment, and migration during the recellularization process [13, 38–41]. In a similar study on the recellularization of the heart acellular scaffold, the effect of bFGF on cell attachment, proliferation, differentiation, and migration in the heart acellular scaffold was investigated. The results demonstrated that the cell attachment increased significantly in the group in which bFGF was attached to the scaffold using the EDC-NHS linker compared to the passive bFGF attachment group, which did not use the EDC-NHS linker [13].

In addition, a limited number of studies investigated the cytotoxic effects of detergent remnants in the decellularized scaffold on cell viability during the recellularization process. Pu et al. studied the cytotoxic effect of detergents such as TX and the impact of repeated PBS washes on cell viability [5]. The authors reported that TX has a highly cytotoxic effect on human umbilical cord mesenchymal stem cells, which can be reduced by increasing the number of PBS wash cycles; however, this cytotoxic effect is not eliminated and has a detrimental influence on the quality of the recellularization process [5]. To the best of the authors’ knowledge and based on the literature review, this is the first study to use Bio-Beads SM-2 in order to absorb detergent remnants from the acellular scaffold to increase cell viability and proliferation within the scaffold. The results revealed that washing the scaffold using Bio-Beads SM-2 and PBS significantly increased the viability of cells compared to washing the scaffold with PBS alone. Therefore, it is suggested that utilizing Bio-Beads SM-2 as an absorptive agent for detergents could be a novel and promising approach toward cell viability augmentation and improving recellularization quality.

Regarding the recellularization quality, proper seeding of endothelial cells in tunica intima has great importance in the prevention of the exposure of tissue ECM to blood flow, platelet aggregation, and thrombosis. Moreover, ECM exposure to blood flow leads to the stimulation of inflammatory cells, internal layer hyperplasia, and vascular lumen constriction [42]. Accordingly, coherent seeding of endothelial cells during the recellularization process is crucial for the prevention of thrombosis and inflammatory responses. Several studies have investigated the effect of growth factors and some biomolecules such as heparin, VEGF, granulocyte-colony stimulating factor, brain-derived growth factor, and stromal cell-derived factor on efficient endothelialization strategies [43–48]. In the present study, cell attachment to the acellular scaffold increased significantly in the presence of apelin-13. Furthermore, in this group, endothelial cells were placed more uniformly on the vascular lumen surface, which resulted in reducing the risk of thrombosis. On the other hand, vascular smooth muscle and fibroblast cells were placed in the external vascular layer, which was possibly due to the long time needed for cell migration. This finding is also consistent with the results of the study by Qunit et al., in which they recellularized an acellular vessel in vitro using endothelial cells, and then transplanted it to a porcine; however, there was no evidence of VSMC migration toward the tunica media and the cells were halted in the tunica adventitia despite the proper endothelialization of the tunica intima after 30 days of follow-up [24]. Briefly, despite the advancements in decellularization techniques, developing tissue-engineered blood vessels using decellularization and recellularization processes is still facing miscellaneous challenges, which need to be addressed before commercialization.

## Conclusions

In conclusion, this study proposed 16 h of perfusion with 0.25% SDS + 0.5% TX as a promising decellularization approach, which requires little preparation time for transplantation, eliminates cell components, and preserves the ECM content efficiently. Moreover, we introduced a novel approach, in which 1 g/mL Bio-Bead SM-2 was perfused to the acellular vessel for 48 h to remove the remnants of detergents. The results indicated increased cell viability within the scaffold and accelerated acellular tissue preparation. Furthermore, the FESEM and fluorescent imaging revealed that by coating the acellular scaffold with apelin-13, the efficiency of recellularization improved significantly in terms of cell attachment, proliferation, and uniform cell distribution within the lumen. Accordingly, the use of apelin-13 bioconjugation is suggested in prospective, in vivo, and clinical trial studies, and it is proposed that acellular vessels coated with apelin-13 can be commercialized and sold as promising candidates for vascular transplantation.

## Data Availability

The data that support the findings of this study are available from the corresponding authors upon reasonable request.

## References

[1] Mathers CD, Loncar D (2006). Projections of global mortality and burden of disease from 2002 to 2030. PLoS Med.

[2] Kuna VK, Xu B, Sumitran-Holgersson S (2018). Decellularization and Recellularization methodology for human saphenous veins. JoVE (Journal of Visualized Experiments).

[3] Rambøl MH, Hisdal J, Sundhagen JO, Brinchmann JE, Rosales A (2018). Recellularization of decellularized venous grafts using peripheral blood: a critical evaluation. EBioMedicine.

[4] Franco C, Gerhardt H (2012). Blood vessels on a chip. Nature.

[5] Pu L, Wu J, Pan X, Hou Z, Zhang J, Chen W (2018). Determining the optimal protocol for preparing an acellular scaffold of tissue engineered small-diameter blood vessels. J Biomed Mater Res B Appl Biomater.

[6] Badylak SF, Taylor D, Uygun K (2011). Whole-organ tissue engineering: decellularization and recellularization of three-dimensional matrix scaffolds. Annu Rev Biomed Eng.

[7] Heine J, Schmiedl A, Cebotari S, Mertsching H, Karck M, Haverich A (2011). Preclinical assessment of a tissue-engineered vasomotive human small-calibered vessel based on a decellularized xenogenic matrix: histological and functional characterization. Tissue Eng A.

[8] Guyette JP, Gilpin SE, Charest JM, Tapias LF, Ren X, Ott HC (2014). Perfusion decellularization of whole organs. Nat Protoc.

[9] Crapo PM, Gilbert TW, Badylak SF (2011). An overview of tissue and whole organ decellularization processes. Biomaterials..

[10] Ullah M, Wahab A, Khan D, Saeed S, Khan SU, Ullah N (2021). Modified gold and polymeric gold nanostructures: toxicology and biomedical applications. Colloid Interface Sci Commun.

[11] Ketchedjian A, Jones AL, Krueger P, Robinson E, Crouch K, Wolfinbarger L (2005). Recellularization of decellularized allograft scaffolds in ovine great vessel reconstructions. Ann Thorac Surg.

[12] Hsia K, Lin C-H, Lee H-Y, Chen W-M, Yao C-L, Chen C-C (2019). Sphingosine-1-phosphate in endothelial cell recellularization improves patency and endothelialization of decellularized vascular grafts in vivo. Int J Mol Sci.

[13] Rajabi S, Pahlavan S, Ashtiani MK, Ansari H, Abbasalizadeh S, Sayahpour FA (2018). Human embryonic stem cell-derived cardiovascular progenitor cells efficiently colonize in bFGF-tethered natural matrix to construct contracting humanized rat hearts. Biomaterials.

[14] Wysocka MB, Pietraszek-Gremplewicz K, Nowak D (2018). The role of apelin in cardiovascular diseases, obesity and cancer. Front Physiol.

[15] Yang X, Zhu W, Zhang P, Chen K, Zhao L, Li J (2014). Apelin-13 stimulates angiogenesis by promoting cross-talk between AMP-activated protein kinase and Akt signaling in myocardial microvascular endothelial cells. Mol Med Rep.

[16] Yang Y, Lv S-Y, Ye W, Zhang L (2016). Apelin/APJ system and cancer. Clin Chim Acta.

[17] Hou J, Zhong T, Guo T, Miao C, Zhou C, Long H (2017). Apelin promotes mesenchymal stem cells survival and vascularization under hypoxic-ischemic condition in vitro involving the upregulation of vascular endothelial growth factor. Exp Mol Pathol.

[18] Tempel D, de Boer M, van Deel ED, Haasdijk RA, Duncker DJ, Cheng C (2012). Apelin enhances cardiac neovascularization after myocardial infarction by recruiting aplnr+ circulating cells. Circ Res.

[19] Lu Q, Jiang Y-r, Qian J, Tao Y (2013). Apelin-13 regulates proliferation, migration and survival of retinal Müller cells under hypoxia. Diabetes Res Clin Pract.

[20] Eyries M, Siegfried G, Ciumas M, Montagne K, Agrapart M, Lebrin F (2008). Hypoxia-induced apelin expression regulates endothelial cell proliferation and regenerative angiogenesis. Circ Res.

[21] Masoud AG, Lin J, Azad AK, Farhan MA, Fischer C, Zhu LF (2019). Apelin directs endothelial cell differentiation and vascular repair following immune-mediated injury. J Clin Invest.

[22] Smith S, Goodge K, Delaney M, Struzyk A, Tansey N, Frey M (2020). A comprehensive review of the covalent immobilization of biomolecules onto electrospun nanofibers. Nanomaterials.

[23] Pugliese R, Gelain F (2020). Cross-linked self-assembling peptides and their post-assembly functionalization via one-pot and in situ gelation system. Int J Mol Sci.

[24] Quint C, Kondo Y, Manson RJ, Lawson JH, Dardik A, Niklason LE (2011). Decellularized tissue-engineered blood vessel as an arterial conduit. Proc Natl Acad Sci.

[25] Simsa R, Padma AM, Heher P, Hellström M, Teuschl A, Jenndahl L (2018). Systematic in vitro comparison of decellularization protocols for blood vessels. PLoS One.

[26] Miyada D, Tappel A (1956). Colorimetric determination of hydroxyproline. Anal Chem.

[27] Saleh TA (2011). The influence of treatment temperature on the acidity of MWCNT oxidized by HNO3 or a mixture of HNO3/H2SO4. Appl Surf Sci.

[28] Ravi S, Chaikof EL (2010). Biomaterials for vascular tissue engineering. Regen Med.

[29] Xu J, Shi G-P (2014). Vascular wall extracellular matrix proteins and vascular diseases. Biochimica et Biophysica Acta (BBA)-Molecular Basis of Disease.

[30] Lin C-H, Hsia K, Ma H, Lee H, Lu J-H (2018). In vivo performance of decellularized vascular grafts: a review article. Int J Mol Sci.

[31] Knight R, Wilcox H, Korossis S, Fisher J, Ingham E (2008). The use of acellular matrices for the tissue engineering of cardiac valves. Proc Inst Mech Eng H J Eng Med.

[32] Hopper RA, Woodhouse K, Semple JL (2003). Acellularization of human placenta with preservation of the basement membrane: a potential matrix for tissue engineering. Ann Plast Surg.

[33] Faulk DM, Carruthers CA, Warner HJ, Kramer CR, Reing JE, Zhang L (2014). The effect of detergents on the basement membrane complex of a biologic scaffold material. Acta Biomater.

[34] White LJ, Taylor AJ, Faulk DM, Keane TJ, Saldin LT, Reing JE (2017). The impact of detergents on the tissue decellularization process: a ToF-SIMS study. Acta Biomater.

[35] Wagenseil JE, Mecham RP (2012). Elastin in large artery stiffness and hypertension. J Cardiovasc Transl Res.

[36] Novak P, Kruppa GH (2008). Intra-molecular cross-linking of acidic residues for protein structure studies. Eur J Mass Spectrometry.

[37] Liu R, Zhang F, Zuo BQ, Zhang HX (2011). EDC-crosslinked electrospun silk-fibroin fiber mats. Advanced Materials Research. Trans Tech Publ.

[38] Liu J, Liu M, Chen L (2017). Novel pathogenesis: regulation of apoptosis by Apelin/APJ system. Acta Biochim Biophys Sin.

[39] Plein A, Fantin A, Denti L, Pollard JW, Ruhrberg C (2018). Erythro-myeloid progenitors contribute endothelial cells to blood vessels. Nature.

[40] Kunduzova O, Alet N, Delesque-Touchard N, Millet L, Castan-Laurell I, Muller C (2008). Apelin/APJ signaling system: a potential link between adipose tissue and endothelial angiogenic processes. FASEB J.

[41] Li L, Li L, Xie F, Zhang Z, Guo Y, Tang G (2013). Jagged-1/Notch3 signaling transduction pathway is involved in apelin-13-induced vascular smooth muscle cells proliferation. Acta Biochim Biophys Sin.

[42] Heyligers J, Arts C, Verhagen H, Groot PG, Moll F (2005). Improving small-diameter vascular grafts: from the application of an endothelial cell lining to the construction of atissue-engineered blood vessel. Ann Vasc Surg.

[43] Choi WS, Joung YK, Lee Y, Bae JW, Park HK, Park YH (2016). Enhanced patency and endothelialization of small-caliber vascular grafts fabricated by coimmobilization of heparin and cell-adhesive peptides. ACS Appl Mater Interfaces.

[44] Row S, Santandreu A, Swartz DD, Andreadis ST (2017). Cell-free vascular grafts: recent developments and clinical potential. Technology.

[45] Smith RJ, Koobatian MT, Shahini A, Swartz DD, Andreadis ST (2015). Capture of endothelial cells under flow using immobilized vascular endothelial growth factor. Biomaterials.

[46] Cho SW, Lim JE, Chu HS, Hyun HJ, Choi CY, Hwang KC (2006). Enhancement of in vivo endothelialization of tissue-engineered vascular grafts by granulocyte colony-stimulating factor. J Biomed Mater Res Part A.

[47] Zeng W, Wen C, Wu Y, Li L, Zhou Z, Mi J (2012). The use of BDNF to enhance the patency rate of small-diameter tissue-engineered blood vessels through stem cell homing mechanisms. Biomaterials.

[48] Yu J, Wang A, Tang Z, Henry J, Lee BL-P, Zhu Y (2012). The effect of stromal cell-derived factor-1α/heparin coating of biodegradable vascular grafts on the recruitment of both endothelial and smooth muscle progenitor cells for accelerated regeneration. Biomaterials.

